# Analysis of diagnostic error cases among Japanese residents using diagnosis error evaluation and research taxonomy

**DOI:** 10.1002/jgf2.388

**Published:** 2021-01-04

**Authors:** Taku Harada, Taiju Miyagami, Takashi Watari, Tetsuya Hiyoshi, Kotaro Kunitomo, Takahiro Tsuji, Taro Shimizu

**Affiliations:** ^1^ Division of General Medicine Showa University Koto Toyosu Hospital Tokyo Japan; ^2^ Diagnostic and Generalist Medicine Dokkyo Medical University Hospital Tochigi Japan; ^3^ Department of General Medicine Juntendo University Faculty of Medicine Tokyo Japan; ^4^ Postgraduate Clinical Training Centre Shimane University Hospital Shimane Japan; ^5^ Department of General medicine Fukuoka University Hospital Fukuoka Japan; ^6^ Department of Internal Medicine Kumamoto Medical Center Kumamoto Japan

**Keywords:** diagnostic error, diagnostic process, diagnostic reasoning

## Abstract

The process of diagnostic errors among Japanese residents has not been previously studied. This descriptive study was conducted in June 2019 on junior residents at a single‐center educational hospital in Japan. Diagnosis Error Evaluation and Research taxonomy was used to measure the process of diagnostic error in the most memorable error cases. High frequency of diagnostic errors resulted from inaccurate/misinterpretation of history, failure/delay in eliciting physical examination findings, inaccurate/misinterpretation of physical examination, failure in weighting of physical examination, and failure/delay in considering the diagnosis. Residents made diagnostic errors mainly during history taking, physical examination, and assessment.

## Graphical abstract

According to the analysis of diagnostic error cases among Japanese residents using Diagnosis Error Evaluation and Research taxonomy, diagnostic errors are mainly caused during medical history collection, physical examination, and clinician evaluation.

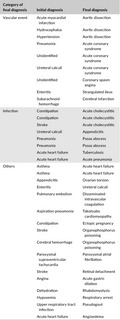

## 1 INTRODUCTION

Diagnostic errors are the number one cause for medical litigation; with an occurrence frequency of 10–15%,^1^ resulting in 40 000–80 000 deaths annually.^2^ Estimated economic loss from unnecessary testing, treatment, and deaths because of diagnostic errors is approximately 30% of the annual total national medical expense.^3^

However, little is known about the actual state of diagnostic errors in Japan because of negative impressions and difficulties conducting research. Previous literature has shown that education about clinical reasoning and the diagnostic process may be lacking in Japan^4^ and that Japanese residents are less knowledgeable about diagnostic errors than are their American residents.^5^ Additionally, since overtesting is very common in Japanese hospitals,^6^ residents can order unnecessary tests without clinical reasoning.^7^

The literature on the diagnostic process among Japanese residents is negligible and not well understood, especially regarding where diagnostic errors are made. Although diagnostic errors are multifactorial, knowing the aspects that are likely to be mistaken during the diagnostic process can help predict areas that are likely to be affected and provide information on which to target interventions. Thus, this study aimed to examine the diagnostic error process among Japanese residents using Diagnosis Error Evaluation and Research (DEER) taxonomy.^8^ The DEER taxonomy is a method developed by Schiff et al. to systematically evaluate diagnostic errors for each diagnostic process; it is easy to use and has moderate inter‐rater reliability.^8^

## 2 METHODS

This descriptive study on junior residents at a single‐center educational hospital was conducted by administering a questionnaire survey to solicit the most memorable diagnostic error cases. The hospital is a regional core hospital with 550 beds and 19 clinical residents per year. The survey was administered to residents during a lecture on diagnostic errors on June 13, 2019. Diagnostic error was defined as any failure in the diagnostic process resulting in a wrong (incorrect diagnosis made before the correct one), missed (no diagnosis was ever made), or unintentionally delayed (diagnosis was not clinically timely) diagnosis.^8^ Physicians provided relevant detailed information on diagnostic error cases, including situational information: location of the occurrence of diagnostic error (emergency visit, outpatient ward, etc.), time zone, chief complaint, presence/absence of examination enforcement, initial and final diagnoses, type of cognitive bias, and type of error. After receiving a brief lecture on cognitive biases in diagnostic errors, physicians reflectively reviewed their cases. The final diagnosis was based on the name of the disease as identified in the post–hospitalization process, feedback, and medical record review. A questionnaire survey on DEER taxonomy was also administered.^8^ In previous studies, physicians chose only one item that contributed the most to the diagnostic errors from among all possible items; in this study, there was an option to select multiple factors that may have contributed to the diagnostic error in order to learn more about the error. Informed consent was obtained from all participants.

## 3 RESULTS

Responses were received from 33 residents (males, 72.7%; mean age, 26.5 ± 1.5 years); 17 and 16 were in the first and second year of graduation, respectively. The questionnaire collection rate was 100%. Of the 33 error cases, 26 occurred in the emergency department and 3 in the ward. For 30 cases, the time of onset was known; 18 occurred during the night shift, 9 in the afternoon, and 3 in the morning. The combination of initial and final diagnoses obtained from the 33 participants is shown in Table 1. Among 33 cases, vascular events (9 cases) and infectious diseases (8 cases) were the main diseases. Table 2 shows the results for the answers to the DEER taxonomy. The most common process that residents believed contributed to diagnostic errors was hypothesis generation (failure/delay in considering the diagnosis) (78.1%); inaccurate/misinterpretation of history (77.4%), inaccurate/misinterpretation of physical examination (71.0%), failure/delay in eliciting critical physical examination finding (67.7%), and failure in weighting of physical examination (61.3%) followed. Residents felt that diagnostic errors occurred during the diagnostic processes of hypothesis generation, physical examination, and history taking when they reflected on their diagnostic error cases.

**Table 1:** List of initial and final diagnoses of 33 cases

Category of final diagnosis	Initial diagnosis	Final diagnosis
Vascular event	Acute myocardial infarction	Aortic dissection
	Hydrocephalus	Aortic dissection
	Hypertension	Aortic dissection
	Pneumonia	Acute coronary syndrome
	Unidentified	Acute coronary syndrome
	Ureteral calculi	Acute coronary syndrome
	Unidentified	Coronary spasm angina
	Enteritis	Strangulated ileus
	Subarachnoid hemorrhage	Cerebral infarction
Infection	Constipation	Acute cholecystitis
	Constipation	Acute cholecystitis
	Stroke	Acute cholecystitis
	Ureteral calculi	Appendicitis
	Pneumonia	Psoas abscess
	Pneumonia	Psoas abscess
	Acute heart failure	Tuberculosis
	Acute heart failure	Acute pneumonia
Others	Asthma	Acute heart failure
	Asthma	Acute heart failure
	Appendicitis	Ovarian torsion
	Enteritis	Ureteral calculi
	Pulmonary embolism	Disseminated intravascular coagulation
	Aspiration pneumonia	Takotsubo cardiomyopathy
	Constipation	Ectopic pregnancy
	Stroke	Organophosphorus poisoning
	Cerebral hemorrhage	Organophosphorus poisoning
	Paroxysmal supraventricular tachycardia	Paroxysmal atrial fibrillation
	Stroke	Retinal detachment
	Angina	Acute gastric dilation
	Dehydration	Rhabdomyolysis
	Hypoxemia	Respiratory arrest
	Upper respiratory tract infection	Pseudogout
	Acute heart failure	Angioedema

**Table 2:**
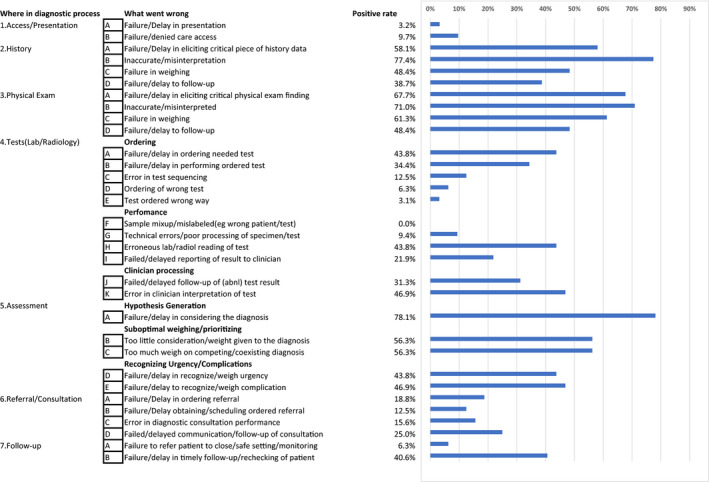
Classification of the cause of diagnostic errors in 33 cases using the diagnostic error evaluation and research taxonomy

## 4 DISCUSSION

This study revealed that when reviewing diagnostic error cases, residents felt that errors mainly occurred during medical history collection, physical examination, and clinician evaluation. In the previous US studies on physicians, DEER items were selected based on being the most influential, with testing phase (44%) and clinician assessment errors (32%) receiving the highest scores.^8^ Specifically, item 5a—failure/delay in considering the diagnosis—was the most common (18%), followed by 4a (11%) and 4h (11%)—failure/delay in ordering the needed test and erroneous laboratory/radiological reading of test, respectively.^8^

This study revealed that medical history, physical examination, and clinician evaluation mainly resulted in diagnostic error cases, which differed from the findings of a study on US clinicians. This may be because of the difference in the selection method of DEER items (single vs. multiple choice) or because, as Tokuda et al. pointed out, there is a lack of education in clinical reasoning based on medical history and physical findings in Japan.^4^ As far as we know, this is the first study of diagnostic errors in Japan using DEER criteria that aimed to elucidate the causes of diagnostic errors among residents.

Despite its novelty, this study has some limitations. First, it is a single‐center study. Because external validity may be poor, we cannot be sure of the trends in Japan as a whole. Second, there are biases in selecting diagnostic error cases, such as biases by facilities, cases diagnosed by residents, information bias, and recall bias. Third, the occurrence of the error in the diagnosis process could change depending on the cases. We must consider this when interventions for diagnostic errors in specific diseases are carried out. Fourth, we did not investigate what types of diagnostic errors or what specific errors were common in the history and physical examination. In future, it is necessary to conduct surveys on a larger scale at multiple facilities.

## 5 CONCLUSION

According to residents, diagnostic errors happened mainly during medical history taking, physical examination, and clinician evaluation.

## CONFLICT OF INTEREST

The authors have stated explicitly that there are no conflicts of interest in connection with this article.

## AUTHOR CONTRIBUTIONS

According to the definition given by the International Committee of Medical Journal Editors (ICMJE), the following individuals qualify for authorship based on their substantial contributions to the manuscript's intellectual content: Taku Harada, Taiju Miyagami, and Takashi Watari for conception and design; Taku Harada for acquisition of data; and Taku Harada for patient management and interpretation of data. Furthermore, Taku Harada, Taiju Miyagami, and Takashi Watari have participated in writing the manuscript. All authors have read and approved the manuscript.

## ETHICAL APPROVAL

The Ethics Committee of Showa University Koto Toyosu Hospital approved the research (No. 19T7009), and written informed consent was obtained from all participants.
